# Effects of different periodontal interventions on the risk of adverse pregnancy outcomes in pregnant women: a systematic review and network meta-analysis of randomized controlled trials

**DOI:** 10.3389/fpubh.2024.1373691

**Published:** 2024-09-20

**Authors:** Jianru Wu, Jingying Wu, Biyu Tang, Ze Zhang, Fenfang Wei, Dingbiao Yu, Limin Li, Yue Zhao, Bei Wang, Wenyu Wu, Xiang Hong

**Affiliations:** ^1^Shenzhen Institute of Pharmacovigilance and Risk Management, Shenzhen, China; ^2^Key Laboratory of Environmental Medicine Engineering, Ministry of Education, School of Public Health, Southeast University, Nanjing, China

**Keywords:** periodontal therapy, intervention measure strategies, adverse pregnancy outcomes, network meta-analysis, randomized controlled trials

## Abstract

**Background:**

Periodontal disease is widespread among pregnant women, and it is possible that taking action to improve oral health conditions can make improvements in adverse pregnancy outcomes. Herein, we summarize the recent evidence using a network meta-analysis to assess the effects of different periodontal treatment intervention strategies on the risk of adverse pregnancy outcomes in pregnant women.

**Materials and methods:**

Randomized controlled trials were retrieved from PubMed, Web of Science, Embase, and Cochrane Library databases. After literature screening, data extraction, and quality evaluation of the included literature were performed, the R studio 4.2.2 “netmeta” package was used for the network meta-analysis.

**Results:**

A total of 20 studies were included, and 5 adverse pregnancy outcomes (preterm birth, low birth weight, preterm birth and/or low birth weight infants, small for gestational age, and pre-eclampsia) were considered to examine the effects of different periodontal treatment interventions strategies on the risk of the abovementioned outcome indicators. The results of the network meta-analysis demonstrated that the three periodontal treatment intervention strategies of sub- and/or supra-gingival scaling and root planing + chlorhexidine rinsing (SRP + CR), sub- and/or supra-gingival scaling and root planing+chlorhexidine rinsing + tooth polishing and plaque control (SRP + CR + TP), and sub- and/or supra-gingival scaling and root planing +sonic toothbrush + tooth polishing and plaque control (SRP + ST + TP) reduced the risk of preterm birth [odds ratio (OR) = 0.29, 95% confidence interval (CI) (0.10–0.88), OR = 0.25, 95CI% (0.10–0.63), OR = 0.28, 95CI% (0.11–0.69), respectively]. In addition, two periodontal treatment intervention strategies, SRP + CR and SRP + CR + TP, were effective methods in terms of the risk of preterm birth and/or low birth weight [OR = 0.18, 95CI% (0.06–0.52), OR = 0.31, 95CI% (0.12–0.79)].

**Conclusion:**

The available evidence suggests that the risk of preterm birth and preterm birth and/or low birth weight can be reduced with certain periodontal treatment intervention strategies. Future studies should focus on optimizing intervention strategies and the optimal timing for different periods of pregnancy, in order to provide a reference for pregnant women’s healthcare.

**Systematic review registration:**

https://www.crd.york.ac.uk/PROSPERO/display_record.php?RecordID=407901, CRD42023407901.

## 1 Introduction

Periodontal disease (PD) is a common global health issue and is a chronic multifactorial inflammatory disease affecting the supporting structures of the teeth and the quality of people’s lives (1, 2). As reported by the Global Burden of Disease Study (2016), PD has become the eleventh most prevalent disease worldwide (3). Epidemiological studies suggest that the prevalence of PD ranges from 20 to 61% (4, 5), and is usually triggered by oral bacteria, starting with changeable plaque and gingival tissue inflammation (gingivitis) and proceeding to irreversible destruction of periodontal tissue support and tooth loss (periodontitis) (6, 7). Currently, periodontal treatment measures are mainly divided into non-surgical and surgical therapies (8). Non-surgical therapies include scaling and root planing, plaque and calculus removal using instruments (scalers and curettes), ultrasound equipment (mechanical debridement including sub-gingival and supra-gingival debridement), and polishing of the teeth (9–13). In addition, routine dental care is also essential, Salzer et al. summarized that a powered toothbrush and use of triclosan dentifrice were better than a manual toothbrush and fluoride-dentifrice in plaque and gingivitis control. In terms of reducing the risk of caries and periodontitis, interdental cleaning with dental floss, interdental brushes, woodsticks, and oral irrigators were effective practices (14). Furthermore, oral hygiene instructions suggest that medications such as metronidazole and doxycycline, and mouthwashes such as chlorhexidine were all non-surgical periodontal treatments (15–17). With regard to severe periodontitis, local or systemic antibiotics and surgical therapies consisting of gingivectomy or flap surgery, etc. should be considered (9, 18). At present, periodontal treatment intervention strategies are used alone or a combination of two or three of the above measures.

The prevalence of PD in pregnant women is approximately 5–40% (19, 20). As the oral conditions of pregnant women have a crucial impact on their own health and the future of their baby (21, 22), increased attention regarding PD in pregnancy has risen over the last few years. According to the World Health Organization (WHO), the prevalence of adverse pregnancy outcomes (APOs) remains high despite the advances in prenatal care and strengthened public awareness. APOs have been defined as (a) pre-term birth when there is delivery before 37 completed weeks (<259 days); (b) pre-eclampsia, which is a multisystem disorder of pregnancy characterized by maternal hypertension and proteinuria after the 20th gestational week; (c) low and very low birth weight, depending on whether the weight of the baby is less of 2,500 g or < 1,500 g; and (d) the spontaneous death of the fetus with <20 weeks (miscarriage) or between 20 and 36 weeks (stillbirth) (23).

Studies have shown that APOs involving preterm birth, low birth weight, pre-eclampsia, gestational diabetes, and perinatal fatality may be linked to poor oral health (24). Previous mechanistic studies provided evidence that the transfer of periodontal pathogens and inflammatory mediators from the infected periodontal ligament or pocket to the fetal and placental unit may trigger an inflammatory cascade response and metastatic infection (25). Owing to the special nature of pregnant women with higher levels of estrogen and progesterone, pregnant women are more likely to suffer from PD caused by a variety of factors (26). However, the degree and mechanisms by which metastatic inflammation and detriment result in APOs and whether the above treatment options are appropriate for pregnant women remain unclear. Hence, the treatment of PD in pregnant women is a topic of concern in dentistry and in obstetrics and gynecology.

At present, there is little evidence from randomized controlled trials (RCTs) that periodontal treatments are safe in pregnant women and can reduce the risk of APOs, such as preterm birth and low birth weight, alleviate PD, and change the condition of gingival crevicular fluid inflammatory mediators (27–35). However, some studies do not agree with this opinion and assert that treatment of periodontitis in pregnant women does not significantly alter the rates of preterm birth, low birth weight, fetal growth restriction, preterm low birth weight, or pre-eclampsia (36–42).

Within recent years, classical meta-analyses have been performed to compare the association between periodontal treatments and pregnancy outcomes in pregnant women. Studies have shown that periodontal treatments during pregnancy have the potential to reduce the incidence of APOs (24, 43, 44). Preventive strategies implemented prenatally have been shown to improve pregnancy outcomes and oral health. Recommendations from health professionals suggest that women can undergo dental screening and treatment interventions during the preconception phase and during pregnancy. However, the association between periodontal treatment interventions and APOs is inconsistent (1). The contradictory results can be attributed to the lack of evidence from face-to-face and high-quality and multicenter trials (RCTs), the inclusion of only a few published studies, and the assessment methods also have incongruities and defects (25). Traditional meta-analyses cannot integrate all the evidence of different treatment interventions at the same time, but network meta-analysis (NMA) manipulates direct and indirect data to contrast interventions (e.g., corresponding to their treatment effects) and distinguish the most effectual options (45).

The principal objective of this project was to further evaluate the effects of different periodontal treatments for pregnant women with PD; an NMA of relevant RCTs was carried out to explicate the effect of different periodontal treatments with respect to the risk of adverse pregnant outcomes and expect to provide the latest evidence upon which to base PD treatment decisions in pregnant women.

## 2 Materials and methods

### 2.1 Study design

This present study is an NMA to assess the effect of different periodontal intervention strategies on adverse pregnancy outcomes. The study population is pregnant women with PDs. The control group consisted of women who did not receive any periodontal treatment during pregnancy but could receive daily dental care, oral hygiene education (OHI), or oral examination (OE). However, the intervention group was given periodontal treatment in addition to the treatments received by the control group. The intervention treatments included sub- and/or supra-gingival scaling and root planing (SRP), tooth polishing and plaque control (TP), extraction of hopeless teeth (ET), chlorhexidine rinsing (CR), adjustment of overhanging restorations (AOR), sonic toothbrush (ST), mouthrinse (containing cetylpyridinium chloride), or metronidazole. The abovementioned single periodontal treatment measures or the combination of two and three treatment measures made up the different periodontal treatment strategies. The outcomes are several adverse pregnancy outcomes, including preterm birth less than 37 weeks (PTB), preterm birth and/or low birth weight (PTLBW), low birth weight less than 2,500 g (LBW), small for gestational age (SGA), preeclampsia (ECL), and abortion and/or stillbirth (AS).

### 2.2 Literature retrieval strategy

RCTs published in English on periodontal treatments in pregnant women were searched in the Cochrane Library, PubMed, Web of Science, and Embase databases. The retrieval period was from inception to 13 October 2022. The study protocol was registered (registration number: CRD42023407901) with the International Prospective Register of Systematic Reviews (PROSPERO). The databases were searched using a combination of subject words and entry terms. We developed a search strategy using the following search terms and their associated medical subject headings to identify all the relevant studies: “periodontal diseases [MeSH Terms],” “periodontal diseases,” “Disease, Periodontal,” “Diseases, Periodontal,” “Periodontal Disease,” “Parodontosis,” “Parodontoses,” “Pyorrhea Alveolaris,” and “Randomized controlled trials.” We modified the search strategy in accordance with the different electronic databases.

### 2.3 Criteria for inclusion and exclusion of studies

Inclusion criteria for the study were as follows: (1) the study was an RCT; (2) the subjects were pregnant women; (3) the control group had periodontal treatment after delivery and the intervention group had periodontal treatment during pregnancy; and (4) at least one outcome indicator in each study can be extracted.

Exclusion criteria for the study were as follows: (1) duplicate publications; (2) articles not relevant to the research topic, such as the outcomes indicators are biomarkers in oral fluids or microbiologic markers; (3) reviews, meta-analyses, *in vitro* studies, and animal experiments; (4) unavailable to obtain the full text, such as experience reports, editorials, letters, conferences, summaries, books, and opinions; and (5) unable to extract the required data or the data were incomplete or could not be transformed into the calculation.

### 2.4 Data extraction and quality assessment

Study characteristics and data from eligible studies were independently extracted by two reviewers (WJY and WJR). If there was disagreement, a third researcher was invited to help solve the problem (HX). The results from each database were imported into EndNote 20 to delete duplicates and initially screen titles and abstracts, and then re-screened by reading the full text according to the inclusion and exclusion criteria to finally determine eligible studies. Basic information included the first author, year of publication, intervention strategies, the sample size of the control group, and intervention group; and the outcomes and the effect sizes were recorded.

Two independent investigators (WJY and WJR) evaluated the methodological quality of the eligible studies by means of the Cochrane Collaboration’s tool for assessing the risk of bias (random sequence generation, allocation concealment, blinding of participants and personnel, blinding of outcome assessment, incomplete outcome data, selective reporting, and other bias) (46). In the event of disagreement between the two authors, a senior investigator was consulted to reach a consensus (HX). Review Manager version 5.4.1 was employed to assess the quality of each eligible study.

Evidence quality of the intervention strategies was rated as high, moderate, low, or very low using the GRADE profiler (GRADEpro) according to the number of studies, study design, risk of bias, inconsistency, indirectness, imprecision, and other considerations. GRADEpro is a computer software application to create summaries of research evidence. It presents key information on all relevant outcomes for a given healthcare question, and a grade for the quality of evidence for each outcome (47, 48).

### 2.5 Statistical analysis

The effect size relative risk (RR) or odds ratio (OR) and its 95% confidence interval (95% CI) of every included study were extracted for the NMA. We calculated the OR and 95%CI using models with random effects and fixed effects as the effect size for comparing different periodontal treatment intervention strategies. The OR was directly pooled across studies using the random effects model if there was heterogeneity, otherwise the fixed effects model was applied. The NMA was performed with the frequentist analysis model by R studio 4.2.2 package “netmeta.” The estimator was based on weighted least-square regression with the Moore–Penrose pseudoinverse method (49). The DerSimonian–Laird random-effects model was used to estimate the variance in heterogeneity between studies (50). A network evidence plot was drawn and the NMA was conducted using the random-effects model. In the network evidence plot, treatment intervention strategies were represented by dots, the size of the dots represented the sample size, and the thickness of the lines between the dots represented the number of direct comparison studies between treatment intervention strategies (51–54).

We introduced the additive component network meta-analysis (CNMA) model into our analysis to compare with the standard NMA model. Generally, in an NMA, existing treatments (single or combined) are different nodes in the network evidence plot. Notwithstanding, there is a situation when we need to utilize the information that treatment intervention strategies consist of elementary active commonplace components. The additive CNMA model enables estimation of the effects of treatment components of combination therapies and a comparison of estimates and model fit among models, which provides a statistical test for the additive model assumption utilizing likelihood ratio statistics. In comparison with the standard NMA model, CNMA models provide more powerful results while having fewer parameters to estimate (number of components instead of number of observations). Additionally, the CNMA model permit strength could be borrowed from studies with common components for combinations that were evaluated in only a handful of studies or only one small study (55). According to a simulation study, the additive effects model is superior to the conventional NMA if the additivity assumption is approximately correct (56). We wanted to introduce this method to identify whether there were differences between these two models in our analysis. Furthermore, intervention strategies were ranked according to the P-score of the frequentist NMA estimate. The P-score is a value based on the point estimates and standard errors with the interpretation of the mean extent of certainty that one intervention strategy was better than another. The higher the P-score, the better the effect of the intervention strategies (57).

## 3 Results

### 3.1 Basic characteristics of retrieved results and quality evaluation

**Figure 1:**
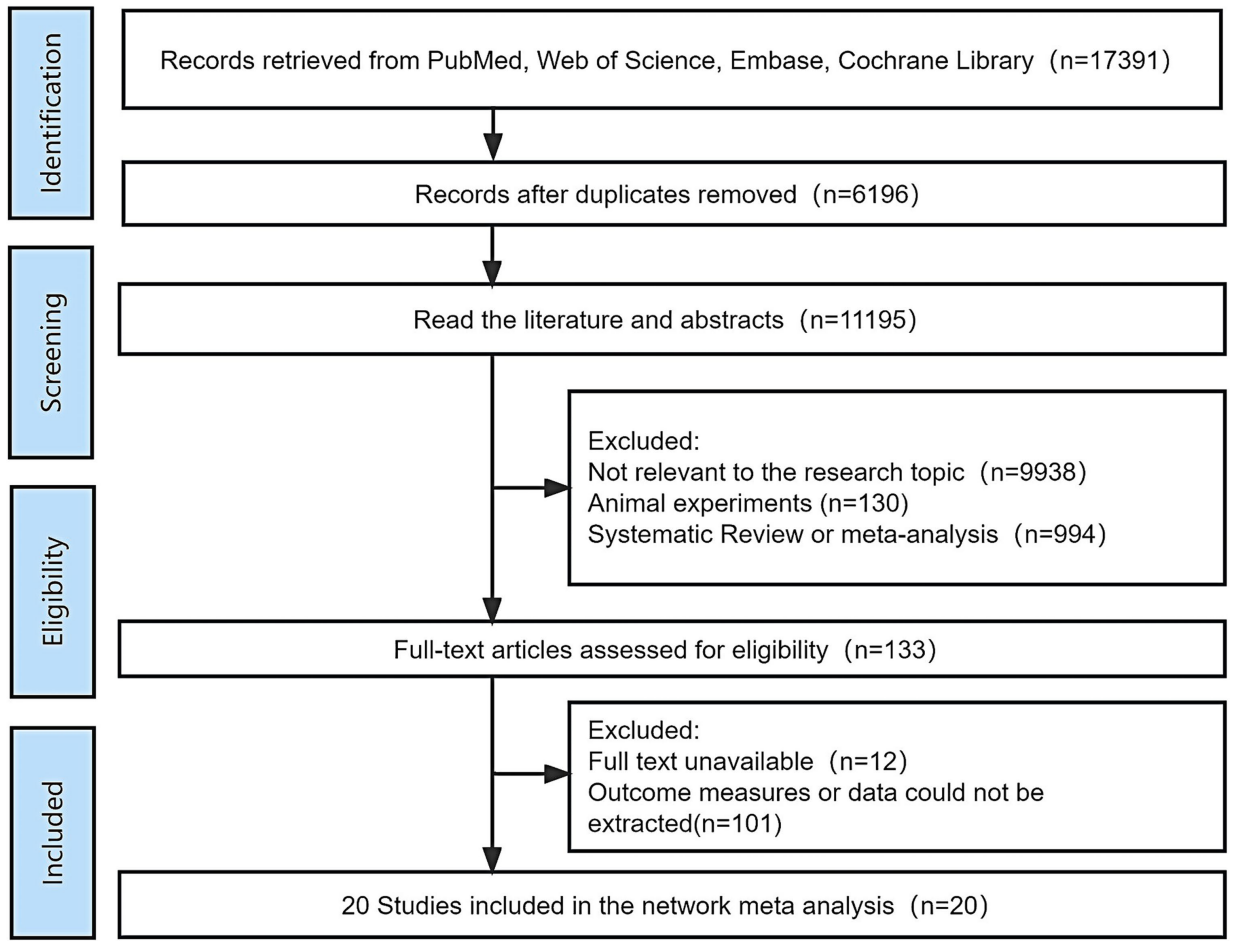
PRISMA flowchart of article selection.

A total of 17,391 articles were retrieved. In total, 11,195 were obtained after importing Endnote 20 to remove 6,196 duplicate articles. A total of 133 articles were then obtained after reviewing the titles and abstracts, manually excluding articles that did not match the topic, and animal experiments, reviews, or meta-analyses. The full texts were carefully read and 20 articles met the inclusion criteria. The flow chart of literature screening is shown in Figure 1.

**Figure 2:**
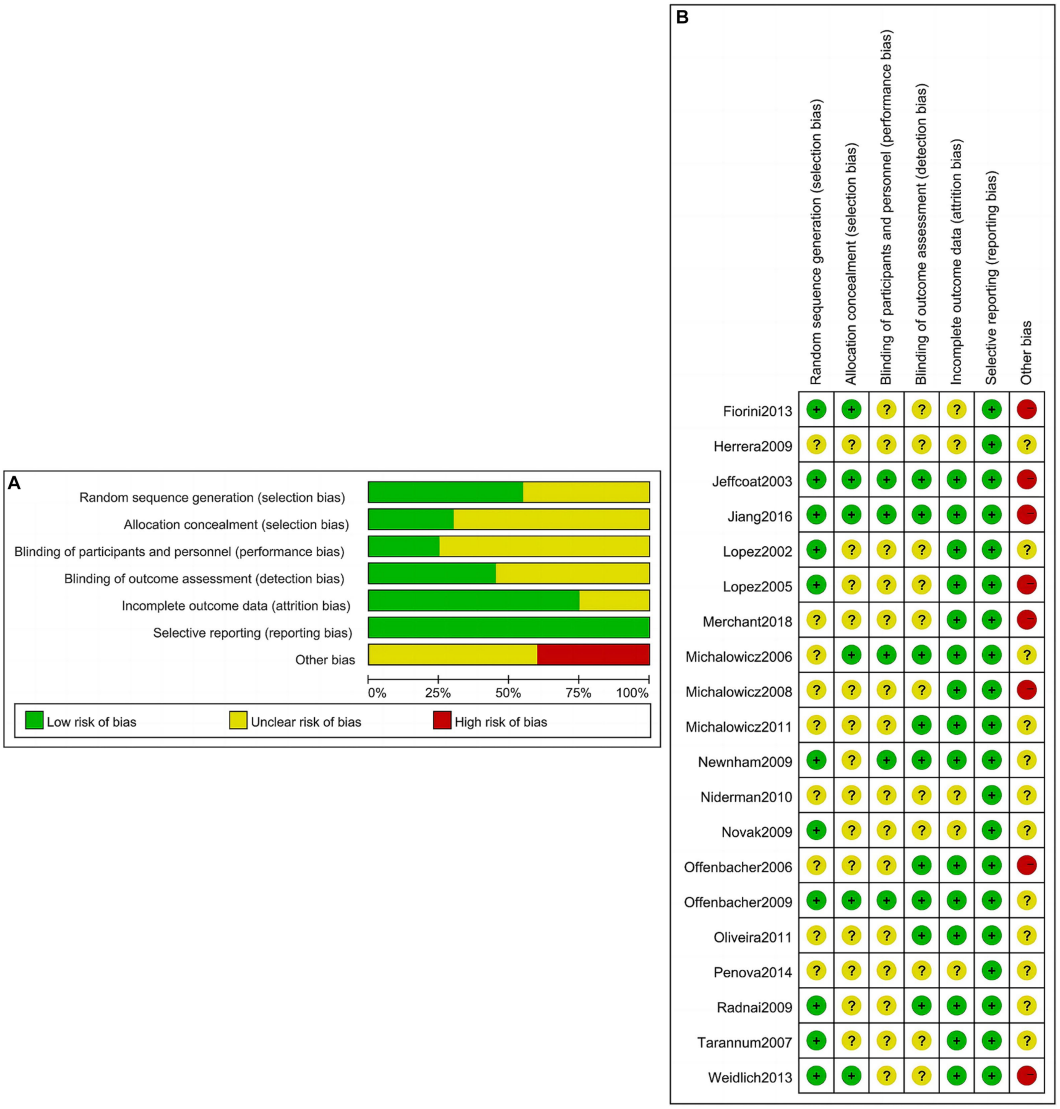
Risk of bias evaluation of included studies. **(A)** Risk of bias graph; **(B)** risk of bias summary.

A total of 20 studies were included in the quantitative NMA, including 5,080 cases in the intervention group and 4,701 cases in the control group as shown in Table 1. One study involved two intervention strategies, and the remaining 19 studies were comparisons between the intervention group and the control group. Outcome measures included preterm birth of less than 37 weeks (PTB), low birth weight of less than 2,500 g (LBW), preterm birth and/or low birth weight (PTLBW), small for gestational age (SGA), eclampsia (ECL), and abortion and/or stillbirth (AS). The quality assessment of the included studies is shown in Figure 2. Of the 20 studies included, merely 10 studies (27–29, 31, 33, 34, 39, 40, 58–60) recorded the unequivocal random grouping methods. Then, the detailed methodology for hiding the random distribution sequences was described in only 6 studies (28, 36, 40, 58–60), leading to a low risk of selection bias. Only two studies (39, 40) executed the blinding methods for research objects and researchers to prevent the disclosure of intervention measures. Moreover, only nine studies (28, 30, 34, 36, 39, 40, 42, 59) implemented the blinding methods for outcome evaluators in an effort to ensure the objectivity of the obtained experimental results. Simultaneously, there was a bias of follow-up in 13 studies (27, 29–32, 34–36, 39, 42, 59–61) but the loss reasons were clearly explained.

**Table 1:** Literature characteristics of included studies in the quantitative analysis.

Number	Included study	Intervention strategies	Control group	Sample size intervention	Sample size control	Outcome indicators
1	Fiorini 2013 (58)	SRP + AOR + ET + TP	Control (OHI)	30	30	PTB
2	Herrera 2009 (38)	SRP	Control	28	32	ECL
3	Jeffcoat 2003 (28)	SRP, SRP + Metronidazole	Control (OHI)	123, 120	123	PTB
4	Jiang 2016 (59)	Mouthrinse	Control (OHI)	232	234	PTB, SGA, LBW
5	Lopez 2002 (27)	SRP + CR	Control (OE)	200	200	PTB, PTLBW, LBW
6	Lopez 2005 (29)	SRP + CR + TP	Control (OHI)	580	290	PTB, PTLBW, LBW
7	Merchant 2018 (35)	SRP	Control (OE)	413	410	PTB, AS
8	Michalowicz 2006 (36)	SRP + TP	Control (OE)	413	410	PTB, SGA, LBW, ECL, AS
9	Michalowicz 2008 (32)	TP	Control (OE)	217	250	PTB, AS
10	Michalowicz 2011 (61)	TP	Control (OE)	205	200	PTB, SGA, LBW
11	Newnham 2009 (39)	SRP + AOR + CR + TP	Control	538	540	PTB, ECL
12	Niderman 2010 (41)	SRP + AOR + TP	Control	542	540	PTB, ECL
13	Novak 2009 (33)	SRP + ST + TP	Control (OE)	44	39	PTB
14	Offenbacher 2006 (30)	SRP + ST + TP	Control (OE)	40	34	PTB, ECL
15	Offenbacher 2009 (40)	SRP + TP	Control	903	903	PTB, SGA, LBW, ECL
16	Oliveira 2011 (42)	SRP	Control	122	124	PTB, PTLBW, LBW
17	Penova 2014 (62)	SRP + AOR + CR + TP	Control (OE)	40	40	PTB, SGA
18	Radnai 2009 (34)	SRP + TP	Control	43	46	PTB, PTLBW, LBW
19	Tarannum 2007 (31)	SRP + CR	Control (OHI)	100	100	PTB, LBW
20	Weidlich 2013 (60)	SRP + AOR + ET + TP	Control (OHI)	147	156	PTB, PTLBW, LBW, ECL, AS

SRP, sub- and supra-gingival scaling and root planing; TP, tooth polishing and plaque control; ET, extraction of hopeless teeth; CR, chlorhexidine rinsing; AOR, adjustment of overhanging restorations; ST, sonic toothbrush; Mouthrinse, containing cetylpyridinium chloride; Metronidazole; Control, the control group received no treatment or oral hygiene instruction (OHI) or oral examination (OE). PTB, preterm birth less than 37 weeks; PTLBW, preterm birth and/or low birth weight; LBW, low birth weight of less than 2,500 g; SGA, small for gestational age; ECL, eclampsia; AS, abortion and/or stillbirth.

### 3.2 Preterm birth

**Figure 3:**
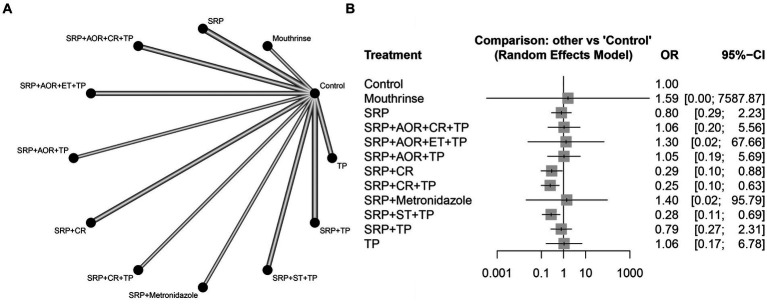
The associations between different periodontal treatment intervention strategies and preterm birth. **(A)** Network evidence plot for preterm birth less than 37 weeks. Each node (black circle) represents a periodontal treatment intervention strategy. The solid lines mean the periodontal treatment intervention strategies of direct comparisons, and the thickness of the lines is proportional to the number of trials. No connecting line between two nodes indicates that there was no direct comparison between the two strategies. **(B)** Forest plot of different periodontal treatment intervention strategies for preterm birth compared to the control group. The data in bold and underlined indicate statistical significance. SRP, sub- and supra-gingival scaling and root planing; TP, tooth polishing and plaque control; ET, extraction of hopeless teeth; CR, chlorhexidine rinsing; AOR, adjustment of overhanging restorations; ST, sonic toothbrush; Mouthrinse: containing cetylpyridinium chloride; Metronidazole; Control.

Among 19 studies (27–36, 39–42, 58–62), the network diagram of 11 intervention strategies for preterm birth less than 37 weeks is presented in Figure 3A. In the network evidence plot, each node (black circle) represents a periodontal treatment intervention strategy. They were mouthrinse, SRP, sub- and supra-gingival scaling and root planing + adjustment of overhanging restorations + chlorhexidine rinsing + tooth polishing and plaque control (SRP + AOR + CR + TP), sub- and supra-gingival scaling and root planing + adjustment of overhanging restorations + extraction of hopeless teeth + tooth polishing and plaque control (SRP + AOR + ET + TP), sub- and supra-gingival scaling and root planing + adjustment of overhanging restorations + tooth polishing and plaque control (SRP + AOR + TP), sub- and supra-gingival scaling and root planing + chlorhexidine rinsing (SRP + CR), sub- and supra-gingival scaling and root planing + chlorhexidine rinsing + tooth polishing and plaque control (SRP + CR + TP), sub- and supra-gingival scaling and root planing + Metronidazole (SRP + Metronidazole), sub- and supra-gingival scaling and root planing + sonic toothbrush + tooth polishing and plaque control (SRP + ST + TP), SRP + TP, and TP. A total of eight periodontal treatment interventions were involved, including SRP, TP, ET, CR, AOR, ST, and mouthrinse containing cetylpyridinium chloride, and metronidazole. The solid lines mean the periodontal treatment intervention strategies of direct comparisons, and the thickness of the lines is proportional to the number of trials. No connecting line between two nodes indicates that there was no direct comparison between the two strategies. The results showed that SRP + TP was included in the largest number of studies compared to the control group (three studies). The network forest plot in Figure 3B shows the OR and 95%CI of all 11 intervention strategies for preterm birth in this NMA. Here, significant differences were found in the following three intervention strategies SRP + CR, SRP + CR + TP, and SRP + ST + TP compared to the control group in terms of reducing the risk of preterm birth less than 37 weeks [OR = 0.29, 95CI% (0.10–0.88), OR = 0.25, 95CI% (0.10–0.63), OR = 0.28, 95CI% (0.11–0.69), respectively]. Other intervention strategies showed no statistically significant association (*p* > 0.05). This means that the abovementioned three intervention strategies may have the potential to reduce the risk of preterm birth.

**Figure 4:**
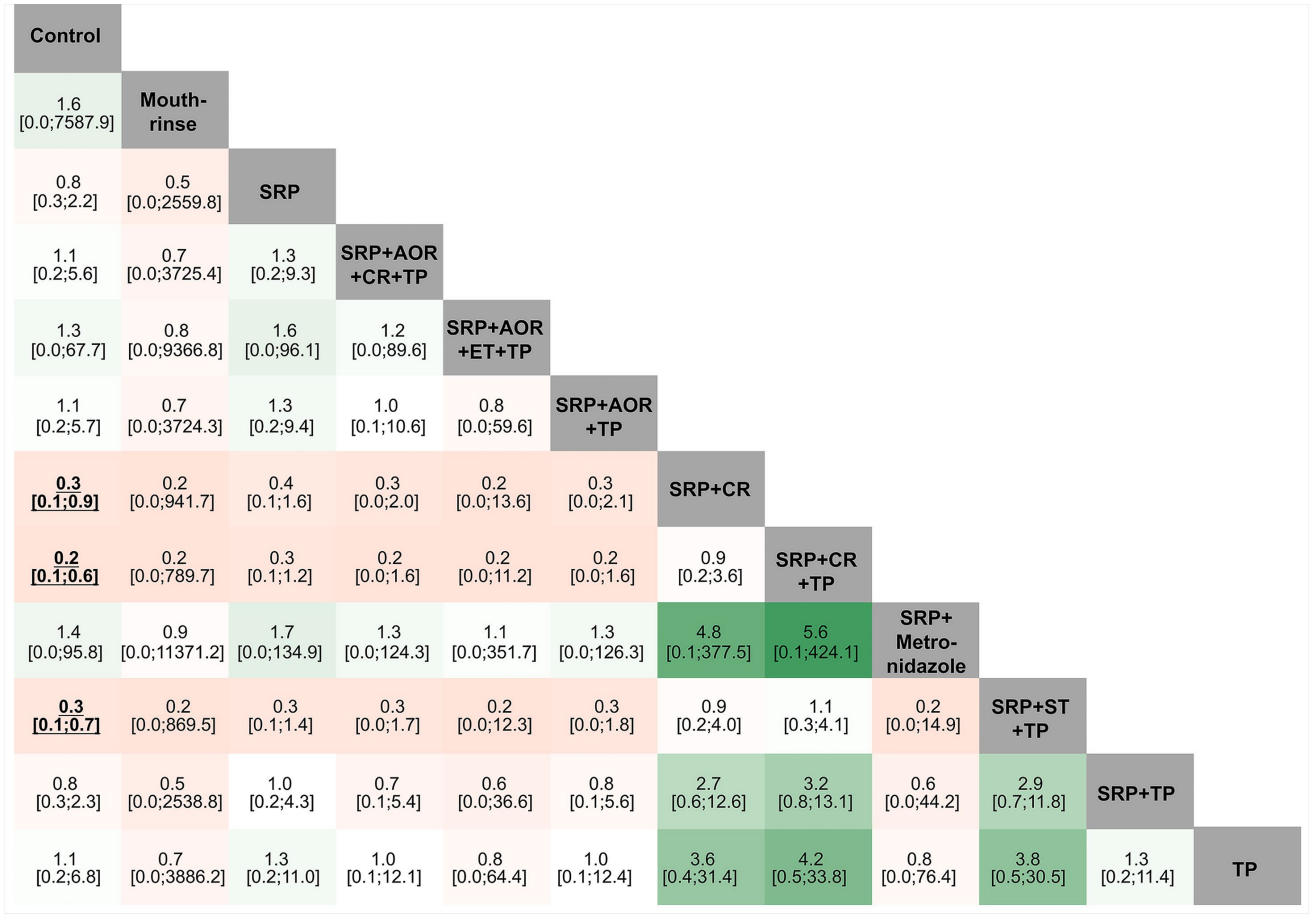
League tables of different periodontal treatment intervention strategies and preterm birth. SRP, Supra- and sub-gingival scaling and root planing; TP, Teeth polishing and plaque control; ET, Extraction of hopeless teeth; CR, Chlorhexidine rinsing; AOR, Adjustment of overhanging restorations; ST, Sonic toothbrush; *Mouthrinse*: containing cetylpyridinium chloride; Metronidazole; Control.

As shown in Figure 4, the league table reflects the relative effects of pairwise comparison between each intervention strategy and control group for the risk of preterm birth (the treatment on the column to the treatment of the row). SRP + CR, SRP + CR + TP, and SRP + ST + TP demonstrated statistically significant results, which indicate that they may reduce the risk of preterm birth (OR and its 95%CI <1) compared to the control group. There was provisionally little evidence of any statistical significance between the remaining pairwise intervention strategies for the risk of preterm birth.

**Figure 5:**
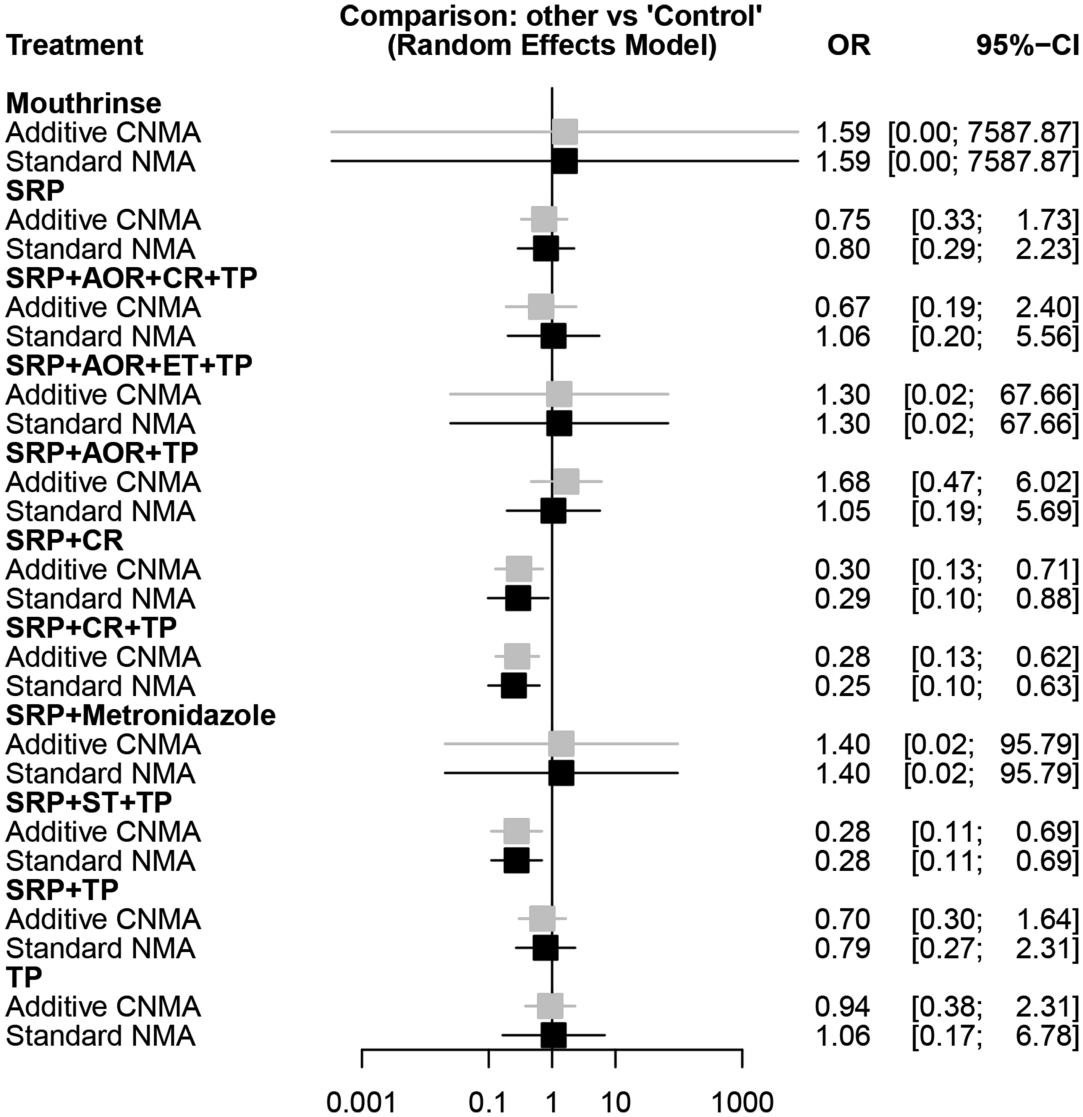
Forest plot of different periodontal treatment intervention strategies and preterm birth less than 37 weeks using the additive CNMA and standard NMA approaches. SRP, Supra- and sub-gingival scaling and root planing; TP, Tooth polishing and plaque control; ET, Extraction of hopeless teeth; CR, chlorhexidine rinsing; AOR, Adjustment of overhanging restorations; ST, Sonic toothbrush; Mouthrinse, containing cetylpyridinium chloride; Metronidazole; Control; CNMA, Component network meta-analysis; NMA, Network meta-analysis.

In the different intervention strategies shown above, there are many common components in the periodontal treatments. Hence, we introduced the additive CNMA model to attempt to discover whether there are differences between the additive CNMA model and the standard NMA model in our analysis. The results showed that these two models had no obvious differences as the values of OR and 95%CI in the two models were very close. In addition, the two models revealed the same results that there were no statistically significant associations between the other intervention strategies and the risk of preterm birth less than 37 weeks except for SRP + CR, SRP + CR + TP, and SRP + ST + TP (Figure 5).

### 3.3 Preterm birth and/or low birth weight

With regard to preterm birth and/or low birth weight, there were five RCTs (27, 29, 34, 42, 60) involving five intervention strategies, including SRP + AOR + ET + TP, SRP + CR, SRP + CR + TP, SRP, and SRP + TP, as shown in the network evidence plot in Supplementary Figure S1A. The forest plot was drawn based on the results of the random-effects model NMA (Supplementary Figure S1B). In contrast to the control group, a significant reduction in preterm birth and/or low birth weight with SRP + CR and SRP + CR + TP intervention strategies was observed [OR = 0.18, 95CI% (0.06–0.52), OR = 0.25, 95CI% (0.12–0.79), respectively]. As shown in the additive CNMA model and the standard NMA model analysis, there were statistically significant associations only for SRP + CR and SRP + CR + TP intervention strategies, while the others showed no statistical significance regarding the risk of preterm birth and/or low birth weight as shown in Supplementary Figure S1C. In addition, the league table reflects the relative effects of pairwise comparison between each intervention strategy and control group for the risk of preterm birth and/or low birth weight (the treatment on the column to the treatment of the row). As shown in Supplementary Figure S1D, SRP + CR, and SRP + CR + TP demonstrated statistically significant results, which indicate that they possibly reduce the possibility of preterm birth and/or low birth weight (OR and 95%CI <1) compared to the control group. The remaining intervention strategies were not statistically significant. Based on these results, the likelihood of preterm birth and/or low birth weight may be reduced when patients receive SRP + CR or SRP + CR + TP intervention strategies.

### 3.4 Low birth weight

With regard to low birth weight less than 2,500 g, there were 10 RCTs (27, 29, 31, 34, 36, 40, 42, 59–61) involving seven intervention strategies, including mouthrinse, SR, SRP + AOR + ET + TP, SRP + CR, SRP + CR + TP, SRP + TP, and TP, as shown in Supplementary Figure S2A. The network evidence plot showed that the number of direct studies between SRP + TP and the control group was greatest (three studies). The forest plot was drawn based on the results of the random-effects model NMA (Supplementary Figure S2B). Compared to the control group, there were no statistically significant results associated with any intervention strategies and the risk of low birth weight less than 2,500 g. Based on the additive CNMA model and the standard NMA model analysis, seven intervention strategies did not differ statistically significantly, as shown in Supplementary Figure S2C. The league table reflects the relative effects of pairwise comparison between each intervention strategy and control group for the risk of low birth weight less than 2,500 g (the treatment on the column to the treatment of the row). As shown in Supplementary Figure S2D, none of the intervention strategies exhibited statistically significant results, which indicated that there was no association between interventions for periodontal treatment and the risk of low birth weight of less than 2,500 g compared to the control group.

### 3.5 Small for gestational age

For small gestational age, there were five RCTs (36, 40, 59, 61, 62) involving four intervention strategies including mouthrinse, SRP, SRP + AOR + CR + TP, SRP + TP, and TP as shown in Supplementary Figure S3A. The network evidence plot showed that there were two direct studies comparing SRP + TP and the control group. The forest plot was sketched based on the results of the random-effects model NMA (Supplementary Figure S3B). Compared to the control group, there were no statistically significant results associated with any intervention strategies and the risk of small for gestational age. Based on the additive CNMA model and the standard NMA model analysis, there were no statistically significant results for the four intervention strategies, as shown in Supplementary Figure S3C. The league table reflects the relative effects of pairwise comparison between each intervention strategy and control group for the risk of small for gestational age (the treatment on the column to the treatment of the row). As shown in Supplementary Figure S3D, none of the intervention strategies exhibited statistically significant results, which indicated that there was no association between the interventions for periodontal treatment and the risk of small gestational age compared to the control group.

### 3.6 Preeclampsia

With regard to pre-eclampsia, there were six RCTs (30, 38–41, 60) involving six intervention strategies, including SRP, SRP + AOR + CR + TP, SRP + AOR + ET + TP, SRP + AOR + TP, SRP + ST + TP, SRP + TP, as shown in the Network evidence plot in Supplementary Figure S4A. The forest plot was sketched based on the results of the random-effects model NMA (Supplementary Figure S4B). Compared to the control group, there were no statistically significant results associated with any intervention strategies and the risk of pre-eclampsia. Based on the additive CNMA model and the standard NMA model analysis, there were no statistically significant results for the six intervention strategies, as shown in Supplementary Figure S4C. Additionally, the league table reflects the relative effects of pairwise comparison between each intervention strategy and control group for the risk of preeclampsia (the treatment on the column to the treatment of the row). As shown in Supplementary Figure S4D, no intervention strategies exhibited statistically significant results, which indicated that there was no association between the interventions for periodontal treatment and the risk of preeclampsia compared to the control group.

### 3.7 Abortion and/or stillbirth

In relation to abortion and/or stillbirth, there were four RCTs (32, 35, 36, 60) involving four intervention strategies, including SRP, SRP + AOR + ET + TP, SRP + TP, and TP, as shown in the network evidence plot in Supplementary Figure S5A. The forest plot was created based on the results of the random-effects model NMA (Supplementary Figure S5B). Compared to the control group, there were no statistically significant results associated with any of the intervention strategies and the risk of abortion and/or stillbirth. Based on the additive CNMA model and the standard NMA model analysis, there were no statistically significant results for the four intervention strategies, as shown in Supplementary Figure S5C. Collectively, the league table reflects the relative effects of pairwise comparison between each intervention strategy and control group for the risk of abortion and/or stillbirth (the treatment on the column to the treatment of the row). As shown in Supplementary Figure S5D, none of the intervention strategies exhibited statistically significant results, which indicated that there was no association between the interventions for periodontal treatment and the risk of abortion and/or stillbirth compared to the control group.

### 3.8 P-scores and grade evidence assessment

To determine the optimal intervention strategies for preventing different APOs, we applied the “netrank” function in the “netmeta” package for analysis as shown in Supplementary Figures S6A–F. It was found that the ranking of each periodontal treatment intervention strategy was different for the different outcome indicators. Taking the example of preterm birth less than 37 weeks, the order was SRP + CR + TP > SRP + ST + TP > SRP + CR > SRP + TP > SRP > mouthrinse > SRP + AOR + ET + TP > SRP + Metronidazole > TP > SRP + AOR + TP > SRP, SRP + AOR + CR + TP > control, which presupposes that SRP + CR + TP is most likely to be the best intervention strategy for preterm birth less than 37 weeks. In addition, the order for preterm birth and/or low birth weight was SRP + CR > SRP + CR + TP > SRP + TP > SRP + AOR + ET + TP > SRP > control. It is deduced that SRP + CR is perhaps the best intervention strategy for PTLBW. Other outcome indicators are not mentioned as they were not statistically significant. The GRADEpro tool is used to judge and categorize the evidence quality of different intervention strategies for APOs. With regard to preterm birth, the results of Grade assessment of the different periodontal treatment intervention strategies are very low or low. The full assessment is detailed in Supplementary Table S1. As for the sensitivity analyses, we eliminated two studies with lower research quality, re-performed the NMA, and drew the forest map. Compared to the combined effect before elimination, the results were similar and relatively stable for the preterm birth outcome indicator (see Supplementary Figure S7).

## 4 Discussion

A total of 20 RCTs on the effect of different periodontal treatment intervention strategies on APOs in women during pregnancy were included in this study. Following an NMA, the results showed a statistically significant association between different periodontal treatment intervention strategies and preterm birth at less than 37 weeks (SRP + CR, SRP + CR + TP, and SRP + ST + TP) and preterm birth and/or low birth weight (SRP + CR and SRP + CR + TP), with no statistically significant association seen in the remaining outcome indicators. In addition, we used the GRADE framework to compartmentalize and exhibit the evidence quality of results and ranked the different intervention strategies, and found that SRP + CR + TP may be the best intervention strategy for preterm birth less than 37 weeks. For preterm birth and/or low birth weight, SRP + CR may be the best intervention strategy.

According to a joint consensus report published by the American Academy of Periodontology and the European Federation of Periodontology in 2013, pregnancy outcomes appear to be adversely affected by periodontal infections, at least in some populations (25). Among the 20 articles included in this study, all the studies showed that periodontal treatment during pregnancy is safe and effective and can improve the periodontal status of pregnant women. Nevertheless, 10 studies concluded that periodontal therapy did not reduce the risk of APOs, yet other studies have suggested a positive effect of periodontal therapy on APOs. As far as the authors are concerned, the reasons for these different results are as follows: (1) the sample sizes of the study populations varied. Some studies were designed with hundreds of individuals, while others included small numbers of people, which may have influenced the inference of the results to some extent; (2) the definition of the control group was dissimilar in each study. After pooling the 20 included studies, 6 studies did not apply any intervention in pregnant women with PD in the control group, while 14 studies involved the intervention of oral examination and oral health education in the control group, which could lead to inconsistent results; (3) the treatments in the intervention group were different in each study. Relevant studies have shown that a single treatment of scaling and root planing or adjunctive metronidazole therapy was ineffective. In this study, 17 studies included SRP in the intervention group, and the vast majority included SRP combined with other treatment measures, which would have resulted in different consequences; (4) the outcomes of the studies were not the same. Most studies focused on preterm birth or small for gestational age as an outcome indicator, while some studies also focused on abortion, preterm birth and/or low birth weight, pre-eclampsia, etc. An analysis of subgroups was conducted on the above APOs for the sake of analyzing the effect and severity of periodontal intervention, which also affected the conclusion.

Since 1990, when Offenbacher’s team found the ability of periodontal bacteria and inflammatory mediators to penetrate the fetal–placental unit through the blood circulation to induce pregnancy complications using a bacteremia model and a “chamber” model (63), the study of the impact of PD on APOs has become a hot topic for scholars. Gradually, a mechanistic model that may explain the biological association between PD and APOs gained general acceptance: First, the direct route, where periodontal pathogens reach and invade the placental tissues of the fetus through hematogenous transmission, triggering ectopic infection (metastatic infection). The presence of these pathogens and/or their causative components in the uterine cavity triggers a localized inflammatory response, causing tissue damage (metastatic injury) and leading to pregnancy complications. Second, it is the indirect route. Inflammatory cytokines and mediators produced by periodontal pathogens at the gingival level activate the systemic inflammatory response after stimulation of the maternal liver via the blood circulation, producing acute precursor reactants. The resulting inflammatory factors and mediators produced by periodontal pathogens, together with those produced by the systemic inflammatory response, accumulate in the uterine cavity, leading to increased intrauterine inflammation (metastatic inflammation), which can lead to pregnancy complications (63–65). To date, it has been established that one of the causes of APOs due to periodontitis is the ectopic colonization of the placenta and entry into the amniotic fluid by periodontopathogenic bacteria via the hemotransfer route from the oral cavity, contacting and infecting the fetus. The evidence for this metastatic route of infection is clear and comes from case report studies, population-based cohort studies, and experimental studies of key PD causative organisms (e.g., *Fusobacterium nucleatum*; *Campylobacter rectus*; *Peptostreptococcus micros*; *Prevotella intermedia*; *Prevotella nigrescens*; *Porphyromonas gingivalis*; *Tannerella forsythia*, and *Treponema denticola*) at the level of cellular and animal models, such as the reviews by Guan et al. (64) and Bobetsis et al. (65). Moreover, with the rapid development of high-throughput genomics, genomics has been used to assess the microbiological status of the placenta, and it has been found that periodontitis has a significant impact on the structure of the placental microbiota and that both Streptococcacea and Mycoplasmataceae have been associated with periodontitis and APOs (66). Another biological mechanism that may be suggestive of APOs due to periodontitis is that intrauterine seeding of oral pathogens may lead to an elevated local immune response, which, combined with induced tissue damage, may lead to APOs. Information on such metastatic injury has been derived mainly from animal model studies (65). Studies have shown that elevated levels of prostaglandin E2 (PGE2), interleukin-1β (IL-1β), interleukin-2 (IL-2), interleukin-6 (IL-6), interleukin-8 (IL-8), interleukin-10 (IL-10), or tumor necrosis factor-*α* (TNF-α) in amniotic fluid or serum are associated with preterm birth (67–71); and that the plasma marker of inflammation C-reactive protein (CRP), is associated with preterm birth, preeclampsia and intrauterine growth restriction (72–74). It has also been found that systemic inflammation caused by periodontal pathogenic bacteria and associated inflammatory mediators through the intrahepatic inflammatory pathway mediated by the blood circulation may be an important causative mechanism of metastatic inflammation, but the evidence to further support this pathway is relatively weak due to the temporary lack of systematic experimental studies (64, 65). In addition, limited case studies suggest that periodontal pathogenic bacteria may enter the amniotic cavity from the oral cavity through the maternal genital tract in an upstream infection, triggering intrauterine infections and leading to pregnancy complications (75, 76). Fetal and maternal metastatic infection and systemic inflammatory responses caused by periodontal pathogens crossing the placental barrier are the underlying pathogenic mechanisms between PD and APOs. However, the presence of pregnant women’s own oral bacteria, the state of her immune system, and the severity of metastatic inflammation leading to APOs; the variety of the oral pathogenic species and their interactions complicate the understanding of the role of periodontal pathogens in pregnancy outcomes; thus, the pathways and causative mechanisms by which PD leads to APOs are not yet fully understood, and the causal association between PD and APOs is currently unclear (25).

By virtue of inconsistencies and drawbacks in study designs, research methods, etc., existing evidence is far from convincing. However, APOs are a major public health issue with significant social and financial implications, coupled with the relatively high incidence of PD in pregnant women and the fact that PD is both preventable and treatable, revealing the potential association of PD with APOs is extremely important for health professionals.

Since its formal introduction by Professor Lumley in 2002 (77), NMA has attracted widespread interest. In recent years, the methodology of NMA has been rapidly developed and has become increasingly sophisticated. Its main advantage is that it allows aggregated analysis and indirect or direct comparison of different interventions for the treatment of the same disease, as well as ranking and visualization of the quantitative effects of outcome indicators. Therefore, we used NMA to investigate the impact of periodontal treatment intervention strategies alone and combined on APOs in women during pregnancy, in order to provide a reference for clinical practice.

This study has some limitations, which are mainly reflected in the following aspects: (1) the outcome indicators were scattered, and the number of articles included for each outcome indicator in the NMA was small, and the included studies are limited in English, which may have led to small sample effect, selectivity bias and publication bias; (2) the level of care in different regions and hospitals, the intensity of the various intervention programs, the severity of the PD suffered by the pregnant woman, the extent to which the pregnant woman will benefit from the treatment she receives, the different periods of pregnancy, the length of time periodontal treatment is given, and the combinations of the various therapeutic interventions may also lead to heterogeneity in the results; (3) limited by the quantity and quality of the included studies, the results should be interpreted and generalized with caution, and the conclusions need to be confirmed by multicenter, large-sample, high-quality collaborative RCTs.

A review of 15 RCT studies based on non-surgical periodontal interventions provided in mid-pregnancy found that periodontal treatment during pregnancy did not remove oral pathogens that had already entered the placenta-fetus in early pregnancy, or did not mitigate the exposure of “key pathogens” that cause intrauterine infections (25). This led some scientists to suggest that periodontal therapy provided in preparation for pregnancy is more effective at preventing placenta–fetal exposure to oral pathogens than interventions during pregnancy (64). Moreover, in addition to external factors such as environment, lifestyle, nutritional conditions, socio-economic factors, genetic background, and exogenous microbial infestation (64), endogenous factors such as the severity of PD, autoimmune status, and the extent to which a pregnant woman benefits from periodontal treatment also influence the occurrence of APOs. If possible, future studies should focus on optimizing periodontal treatment intervention combinations and their timing and investigating their intervention effects and mechanisms on other APOs. For example, questions such as whether there is a difference in the occurrence of APOs between periodontal treatment interventions before and after pregnancy, and between different periods of pregnancy, require scholars to delineate the period of intervention in more detail to explore the associations between the two, so that targeted interventions can be made. Moreover, the long-term maternal and child health outcomes related to periodontal treatment and patient-reported outcomes such as quality of life, satisfaction with treatment, and adherence to periodontal care recommendations during pregnancy are equally valuable outcome indicators that should be considered, so that can provide a more holistic view of the impact of periodontal interventions. Perhaps, some of the limitations should be minimized by performing a sub-analysis. In addition, the findings need to be gelled with clinical experience and individual response in order to provide a comprehensive assessment of the effects of periodontal treatment interventions on APOs in women during pregnancy and to provide a reference for women’s healthcare during pregnancy.

## 5 Conclusion

Summarizing the available evidence, we can conclude that the risk of preterm birth less than 37 weeks, preterm birth, and/or low birth weight can be reduced by certain periodontal treatment intervention strategies. For other outcome indicators, statistically significant associations were not found. In addition, due to the influence of the quality and quantity of the included studies, more high-quality and multicenter RCTs are still required to confirm these findings to ensure the reliability and objectivity of the conclusions.

## Data Availability

The raw data supporting the conclusions of this article will be made available by the authors, without undue reservation.
