# Supplementary material for: Effects of different periodontal interventions on the risk of adverse pregnancy outcomes in pregnant women: a systematic review and network meta-analysis of randomized controlled trials
Source: Front Public Health. 2024 Sep 20;12:1373691. doi: 10.3389/fpubh.2024.1373691 (PMC11449777; doi:10.3389/fpubh.2024.1373691)
Supplement: Supplementary file 1 [file Data_Sheet_1.docx]

**Supporting Information**

**Catalogue**

[**Supporting Information** 1](#_Toc174637513)

[**Figure S1.** The associations between different periodontal treatment intervention strategies and preterm birth and/or low birth weight. 1](#_Toc174637514)

[**Figure S2.** The associations between different periodontal treatment intervention strategies and low birth weight of less than 2500 grams. 2](#_Toc174637515)

[**Figure S3.** The associations between different periodontal treatment intervention strategies and small for gestational age. 3](#_Toc174637516)

[**Figure S4.** The associations between different periodontal treatment intervention strategies and pre-eclampsia. 4](#_Toc174637517)

[**Figure S5.** The associations between different periodontal treatment intervention strategies and abortion and/or stillbirth. 5](#_Toc174637518)

[**Figure S6.** Rank result plot of different periodontal treatment intervention strategies for PTB, PTLBW, LBW, SGA, ECL, AS. 6](#_Toc174637519)

[**Table S1.** Grade assessment of different periodontal treatment intervention strategies for preterm birth less than 37 weeks. 8](#_Toc174637520)

[**Figure S7.** The results of sensitivity analysis for the preterm birth less than 37 weeks outcome. 10](#_Toc174637521)


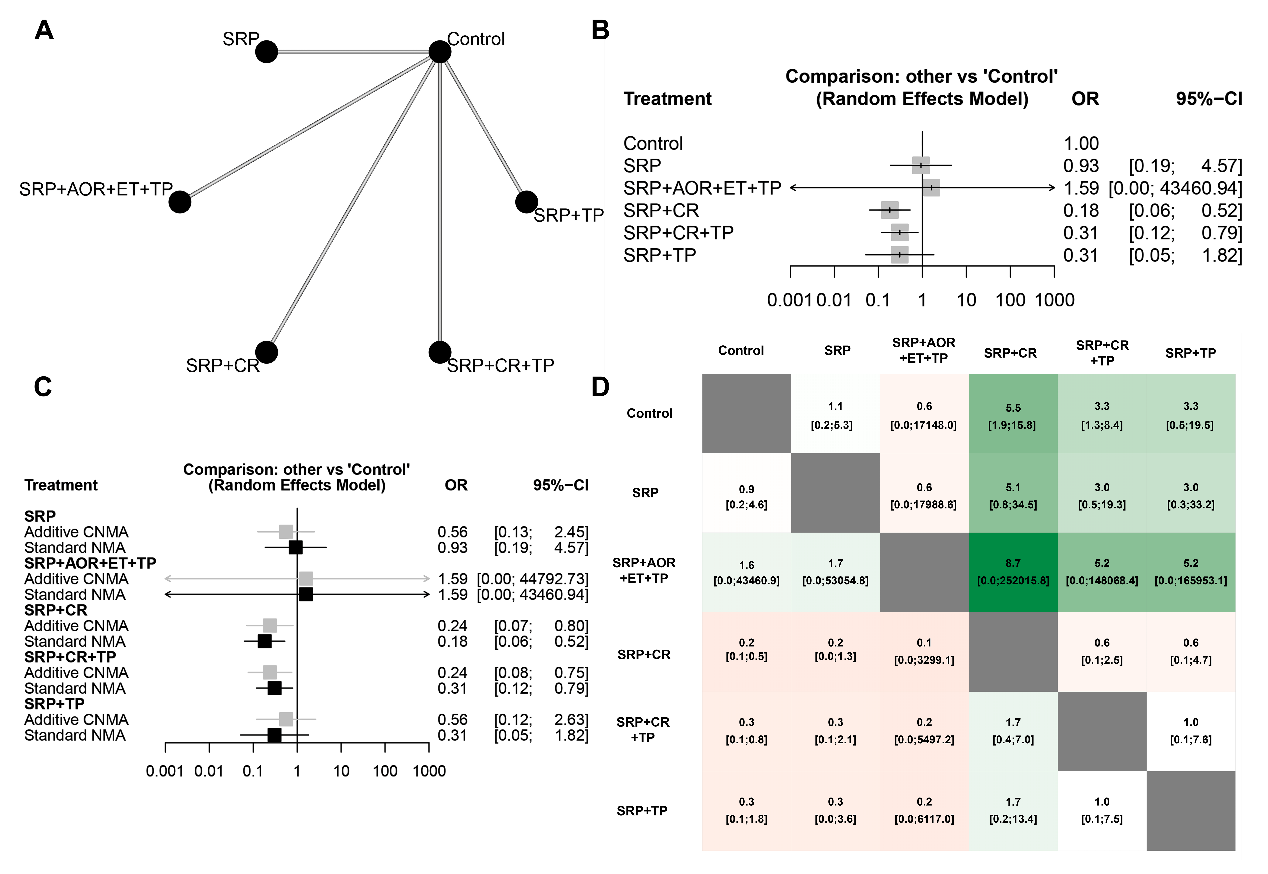


**Figure S1.** **The associations between different periodontal treatment intervention strategies and preterm birth and/or low birth weight.**

(A) Network evidence plot. (B) Forest plot of different periodontal treatment intervention strategies comparing with control group. (C) Forest plot of different periodontal treatment intervention strategies using the additive CNMA and standard NMA approaches. (D) League tables of different periodontal treatment intervention strategies. SRP: sub- and supra- gingival scaling and root planing; TP: tooth polishing and plaque control; ET: extraction of hopeless teeth; CR: chlorhexidine rinsing; AOR: adjustment of overhanging restorations; ST: sonic toothbrush; Mouthrinse: containing cetylpyridinium chloride; Metronidazole; Control: the control group received no treatment or oral hygiene instruction (OHI) or oral examination (OE). PTB: preterm birth less than 37 weeks; PTLBW: preterm birth and/or low birth weight; LBW: low birth weight of less than 2500 g; SGA: small for gestational age; ECL: eclampsia; AS: abortion and/or stillbirth.


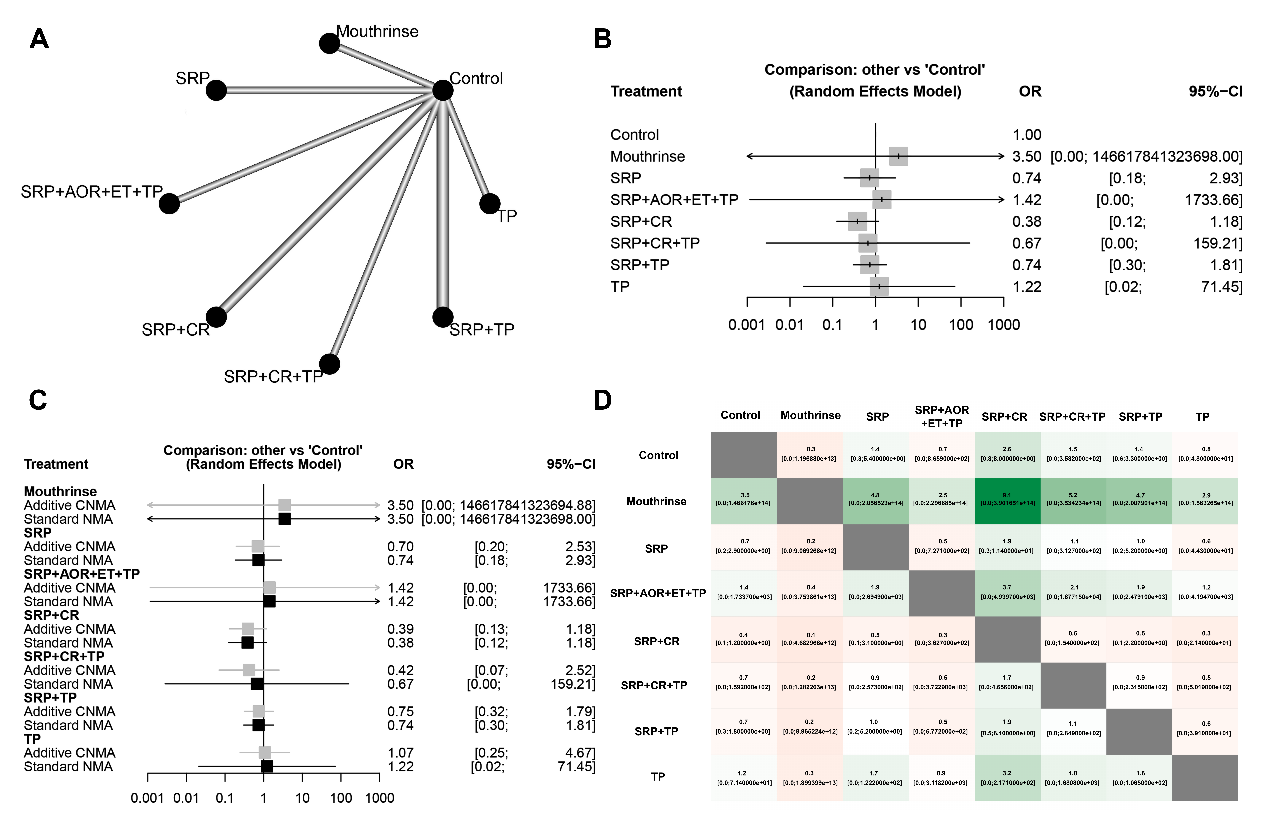


**Figure S2.** **The associations between different periodontal treatment intervention strategies and low birth weight of less than 2500 grams.**

(A) Network evidence plot. (B) Forest plot of different periodontal treatment intervention strategies comparing with control group. (C) Forest plot of different periodontal treatment intervention strategies using the additive CNMA and standard NMA approaches. (D) League tables of different periodontal treatment intervention strategies. SRP: sub- and supra- gingival scaling and root planing; TP: tooth polishing and plaque control; ET: extraction of hopeless teeth; CR: chlorhexidine rinsing; AOR: adjustment of overhanging restorations; ST: sonic toothbrush; Mouthrinse: containing cetylpyridinium chloride; Metronidazole; Control: the control group received no treatment or oral hygiene instruction (OHI) or oral examination (OE). PTB: preterm birth less than 37 weeks; PTLBW: preterm birth and/or low birth weight; LBW: low birth weight of less than 2500 g; SGA: small for gestational age; ECL: eclampsia; AS: abortion and/or stillbirth.


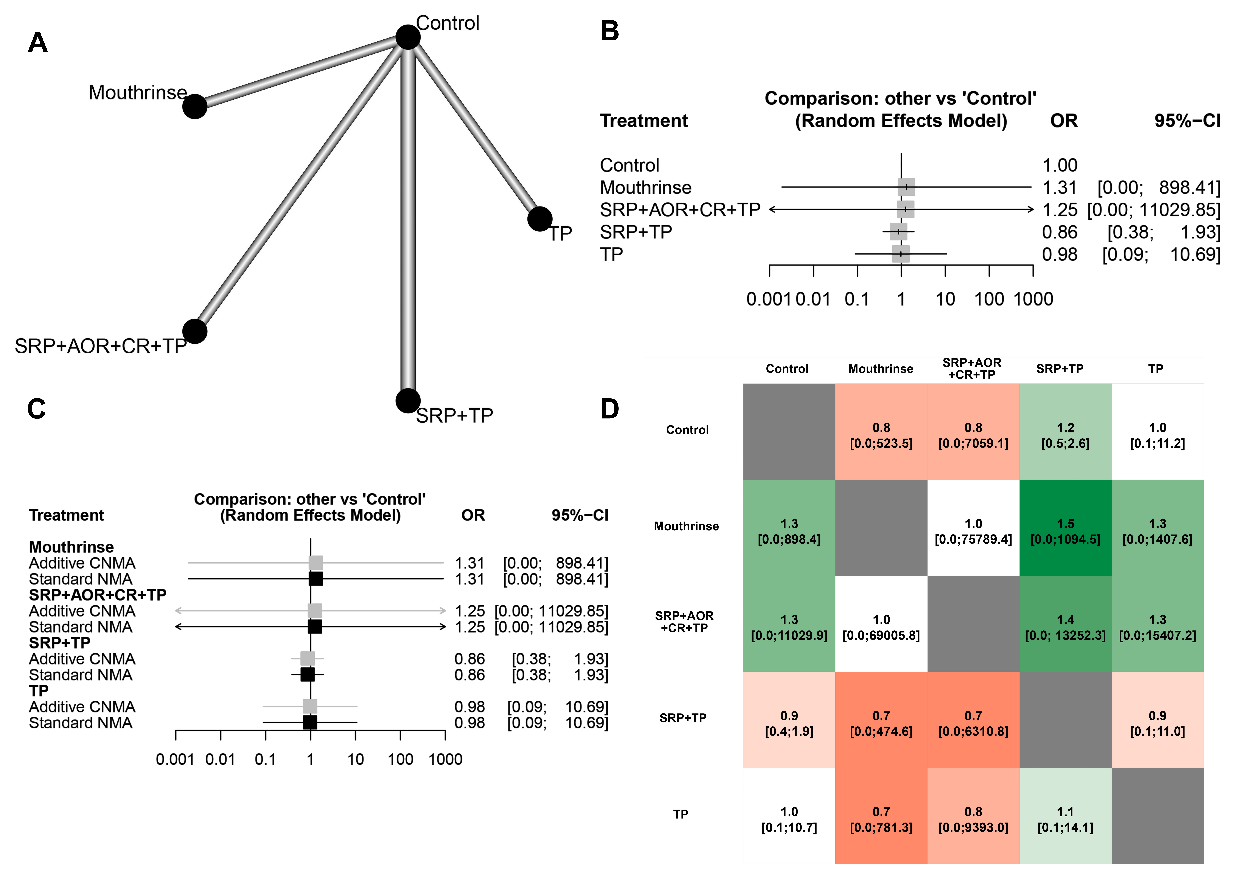


**Figure S3.** **The associations between different periodontal treatment intervention strategies and small for gestational age.**

(A) Network evidence plot. (B) Forest plot of different periodontal treatment intervention strategies comparing with control group. (C) Forest plot of different periodontal treatment intervention strategies using the additive CNMA and standard NMA approaches. (D) League tables of different periodontal treatment intervention strategies. SRP: sub- and supra- gingival scaling and root planing; TP: tooth polishing and plaque control; ET: extraction of hopeless teeth; CR: chlorhexidine rinsing; AOR: adjustment of overhanging restorations; ST: sonic toothbrush; Mouthrinse: containing cetylpyridinium chloride; Metronidazole; Control: the control group received no treatment or oral hygiene instruction (OHI) or oral examination (OE). PTB: preterm birth less than 37 weeks; PTLBW: preterm birth and/or low birth weight; LBW: low birth weight of less than 2500 g; SGA: small for gestational age; ECL: eclampsia; AS: abortion and/or stillbirth.


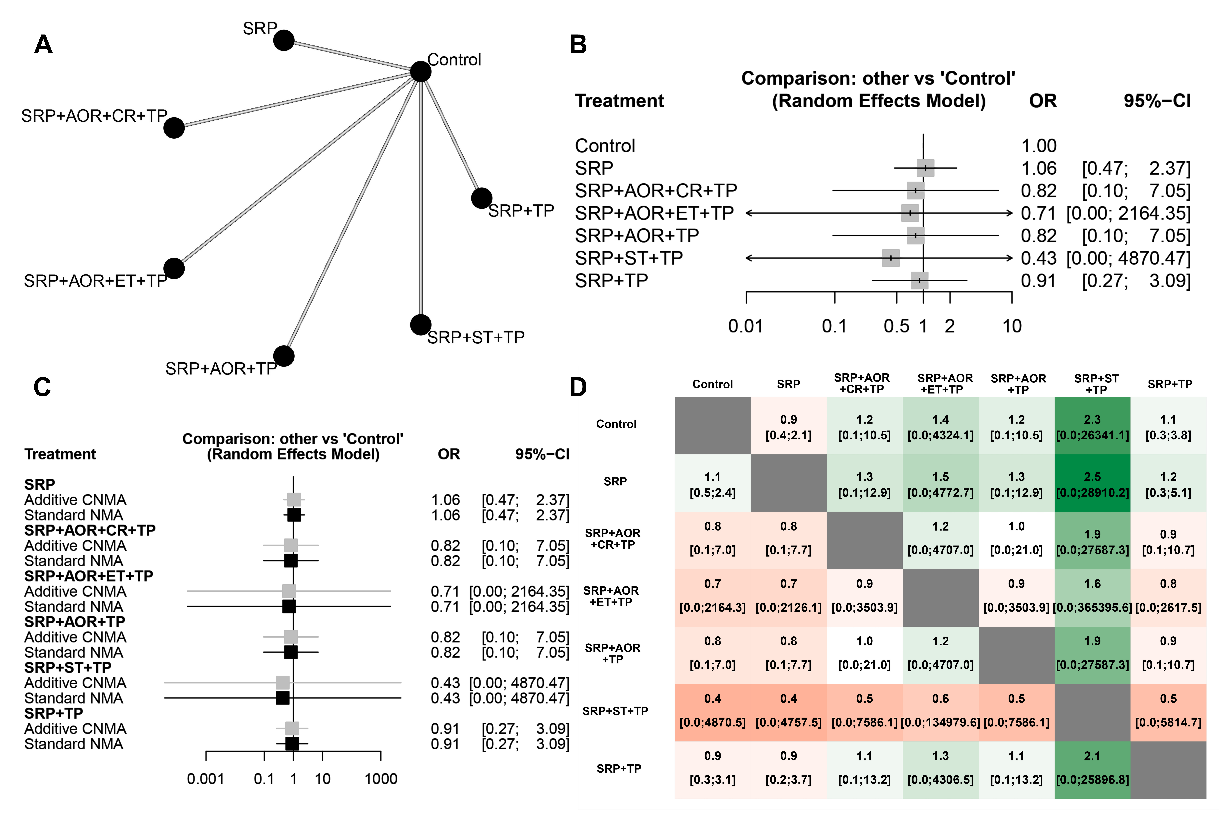


**Figure S4. The associations between different periodontal treatment intervention strategies and pre-eclampsia.**

(A) Network evidence plot. (B) Forest plot of different periodontal treatment intervention strategies comparing with control group. (C) Forest plot of different periodontal treatment intervention strategies using the additive CNMA and standard NMA approaches. (D) League tables of different periodontal treatment intervention strategies. SRP: sub- and supra- gingival scaling and root planing; TP: tooth polishing and plaque control; ET: extraction of hopeless teeth; CR: chlorhexidine rinsing; AOR: adjustment of overhanging restorations; ST: sonic toothbrush; Mouthrinse: containing cetylpyridinium chloride; Metronidazole; Control: the control group received no treatment or oral hygiene instruction (OHI) or oral examination (OE). PTB: preterm birth less than 37 weeks; PTLBW: preterm birth and/or low birth weight; LBW: low birth weight of less than 2500 g; SGA: small for gestational age; ECL: eclampsia; AS: abortion and/or stillbirth.

sf
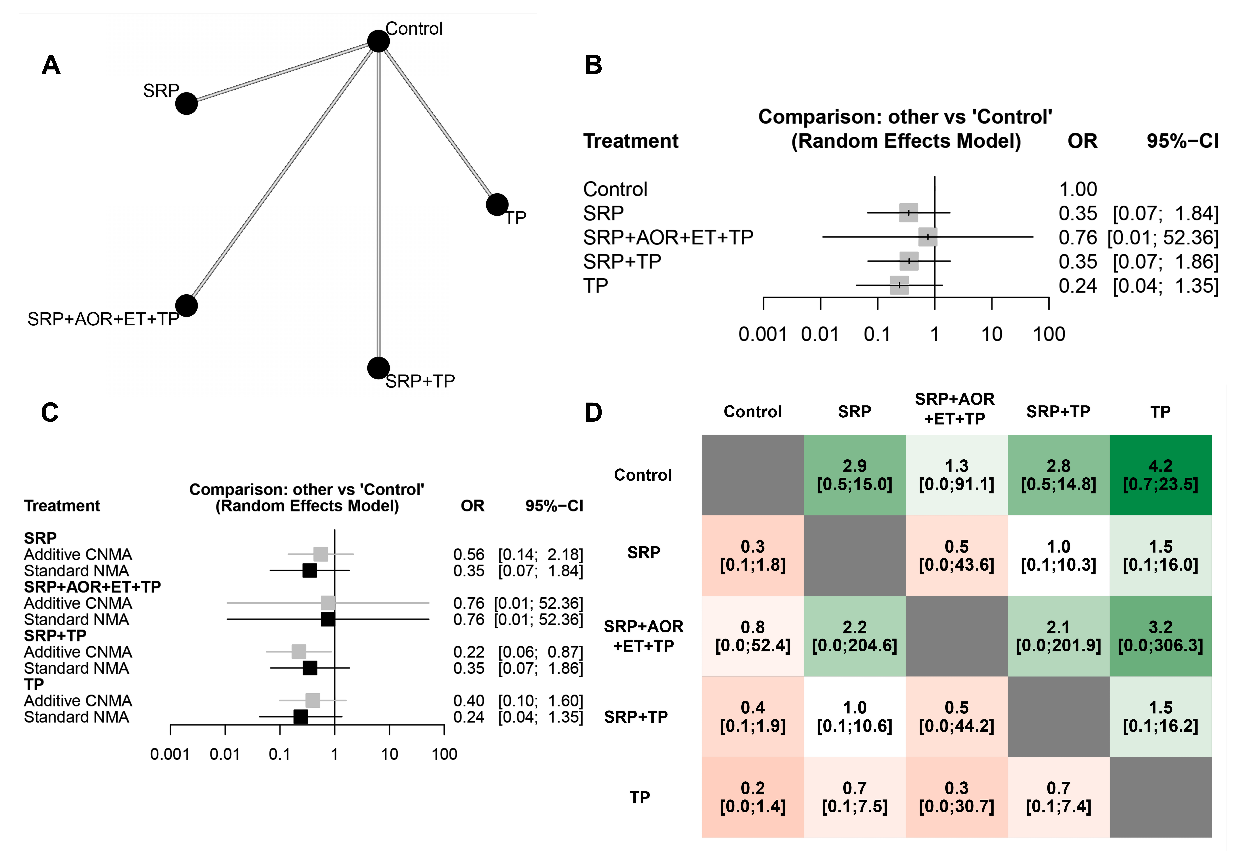


**Figure S5. The associations between different periodontal treatment intervention strategies and abortion and/or stillbirth.**

(A) Network evidence plot. (B) Forest plot of different periodontal treatment intervention strategies comparing with control group. (C) Forest plot of different periodontal treatment intervention strategies using the additive CNMA and standard NMA approaches. (D) League tables of different periodontal treatment intervention strategies. SRP: sub- and supra- gingival scaling and root planing; TP: tooth polishing and plaque control; ET: extraction of hopeless teeth; CR: chlorhexidine rinsing; AOR: adjustment of overhanging restorations; ST: sonic toothbrush; Mouthrinse: containing cetylpyridinium chloride; Metronidazole; Control: the control group received no treatment or oral hygiene instruction (OHI) or oral examination (OE). PTB: preterm birth less than 37 weeks; PTLBW: preterm birth and/or low birth weight; LBW: low birth weight of less than 2500 g; SGA: small for gestational age; ECL: eclampsia; AS: abortion and/or stillbirth.


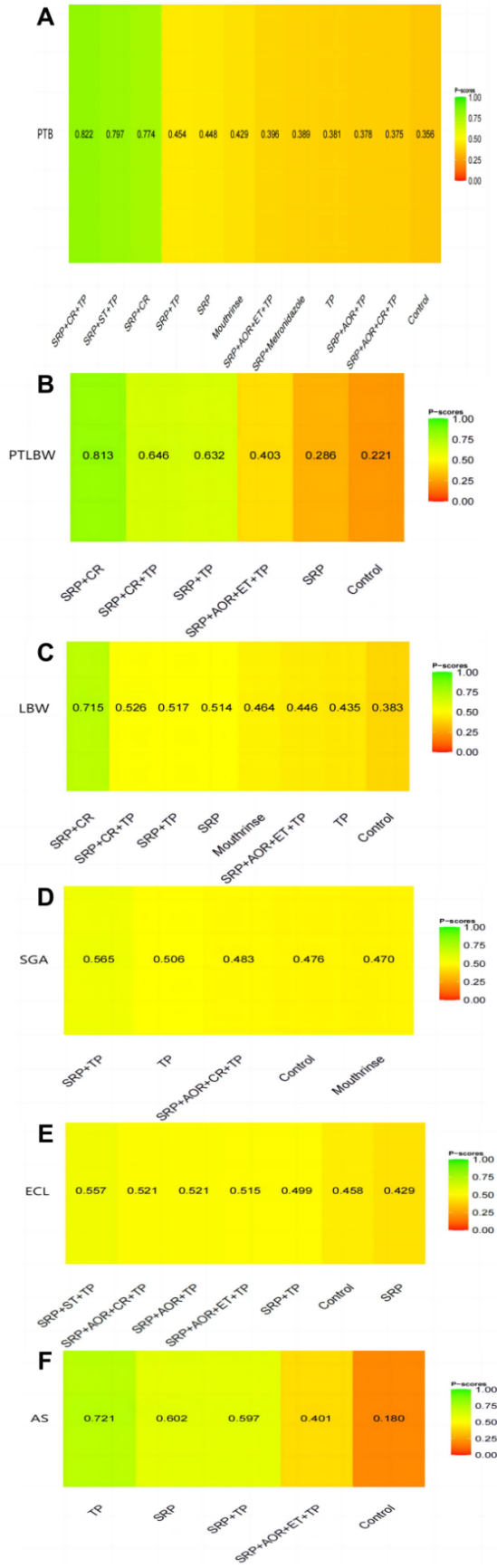


**Figure S6. Rank result plot of different periodontal treatment intervention strategies for PTB, PTLBW, LBW, SGA, ECL, AS.**

(A) PTB: preterm birth less than 37 weeks; (B) PTLBW: preterm birth and/or low birth weight; (C)LBW: low birth weight less than 2500 grams; (D) SGA: small for gestational age; (E) ECL: pre-eclampsia; (F) AS: abortion and/or stillbirth. Higher P-scores indicate better treatments. SRP: sub- and supra- gingival scaling and root planing; TP: tooth polishing and plaque control; ET: extraction of hopeless teeth; CR: chlorhexidine rinsing; AOR: adjustment of overhanging restorations; ST: sonic toothbrush; Mouthrinse: containing cetylpyridinium chloride; Metronidazole; Control: the control group.

**Table S1.** **Grade assessment of different periodontal treatment intervention strategies for preterm birth less than 37 weeks.**

| **Quality assessment** | | | | | | | **No of patients** | | **Effect** | | **Quality** |
| --- | --- | --- | --- | --- | --- | --- | --- | --- | --- | --- | --- |
| **No of**  **studies** | **Design** | **Risk of bias** | **Inconsistency** | **Indirectness** | **Imprecision** | **Other considerations** | **PT** | **Control** | **Relative(95% CI)** | **Absolute** |  |
| **Mouthrinse VS Control** | | | | | | | | | | | |
| 1 | randomised trials | no serious risk of bias | serious^1^ | no serious indi-rectness | very serious^2^ | none | **8/232 (3.4%)** | **5/234 (2.1%)** | OR 1.59 (0 to 7587.87) | 12 more per 1000 (from 21 fewer to 973 more)- | ⊕OOOVERY LOW |
| **SRP VS Control** | | | | | | | | | | | |
| 3 | randomised trials | no serious risk of bias | serious^1^ | no serious indi-rectness | serious^3^ | none | **78/658 (11.9%)** | **89/657 (13.5%)** | OR 0.80 (0.29 to 2.23) | 24 fewer per 1000 (from 92 fewer to 123 more) | ⊕⊕OOLOW |
| **SRP+AOR+CR+TP VS Control** | | | | | | | | | | | |
| 2 | randomised trials | no serious risk of bias | serious^1^ | no serious indi-rectness | serious^3^ | none | **57/578 (9.9%)** | **54/580 (9.3%)** | OR 1.06 (0.2 to 5.56) | 5 more per 1000 (from 73 fewer to 270 more) | ⊕⊕OOLOW |
| **SRP+AOR+ET+TP VS Control** | | | | | | | | | | | |
| 2 | randomised trials | no serious risk of bias | very serious^4^ | no serious indi-rectness | very serious^2^ | none | **20/177 (11.3%)** | **16/186 (8.6%)** | OR 1.30 (0.02 to 67.66) | 23 more per 1000 (from 84 fewer to 778 more) | ⊕OOOVERY LOW |
| **SRP+AOR+TP VS Control** | | | | | | | | | | | |
| 1 | randomised trials | no serious risk of bias | serious^1^ | no serious indi-rectness | serious^3^ | none | **53/542 (9.8%)** | **50/540 (9.3%)** | OR 1.05 (0.19 to 5.69) | 4 more per 1000 (from 74 fewer to 275 more) | ⊕⊕OOLOW |
| **SRP+CR VS Control** | | | | | | | | | | | |
| 2 | randomised trials | no serious risk of bias | no serious inconsistency | serious^5^ | no serious imprecision | none | **55/300 (18.3%)** | **80/300 (26.7%)** | OR 0.29 (0.1 to 0.88) | 171 fewer per 1000 (from 24 fewer to 232 fewer) | ⊕⊕⊕OMODERATE |
| **SRP+CR+TP VS Control** | | | | | | | | | | | |
| 1 | randomised trials | no serious risk of bias | no serious inconsistency | serious^5^ | no serious imprecision | none | **8/580 (1.4%)** | **16/290 (5.5%)** | OR 0.25 (0.1 to 0.63) | 41 fewer per 1000 (from 20 fewer to 49 fewer) | ⊕⊕⊕OMODERATE |
| **SRP+Metronidazole VS Control** | | | | | | | | | | | |
| 1 | randomised trials | no serious risk of bias | very serious^4^ | no serious indi-rectness | very serious^2^ | none | **15/120 (12.5%)** | **11/123 (8.9%)** | OR 1.4 (0.7 to 2.9) | 31 more per 1000 (from 25 fewer to 132 more) | ⊕OOOVERY LOW |
| **SRP+ST+TP VS Control** | | | | | | | | | | | |
| 2 | randomised trials | no serious risk of bias | no serious inconsistency | serious^5^ | no serious imprecision | none | **27/84 (32.1%)** | **33/73 (45.2%)** | OR 0.28 (0.11 to 0.69) | 264 fewer per 1000 (from 89 fewer to 369 fewer | ⊕⊕⊕OMODERATE |
| **SRP+TP VS Control** | | | | | | | | | | | |
| 3 | randomised trials | no serious risk of bias | serious^1^ | no serious ind-irectness | serious^3^ | none | **177/1359 (13%)** | **178/1359 (13.1%)** | OR 0.79 (0.27 to 2.31) | 25 fewer per 1000 (from 92 fewer to 127 more) | ⊕⊕OOLOW |
| **TP VS Control** | | | | | | | | | | | |
| 2 | randomised trials | no serious risk of bias | serious^1^ | no serious indi-rectness | serious^3^ | none | **47/422 (11.1%)** | **48/450 (10.7%)** | OR 1.06 (0.17 to 6.78) | 6 more per 1000 (from 87 fewer to 341 more) | ⊕⊕OOLOW |

^1^ The inconsistency of the study findings was severe, crossing the 1

^2^ The accuracy is not enough, and the credible interval is extremely serious and wide

^3^ Precision is not enough, and the credible interval is seriously wide

^4^ The inconsistency of the study results was extremely severe, crossing 1 and exceeding 0.1 or 10

^5^ It cannot be established that the part is the direct evidence


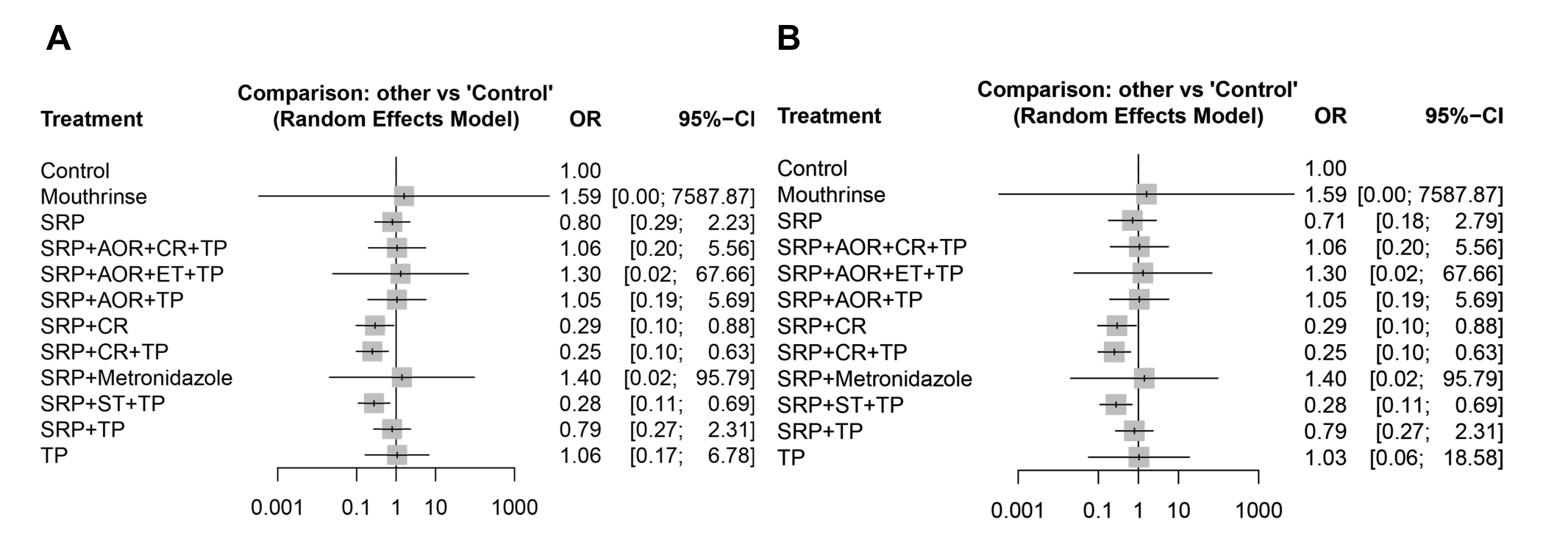


**Figure S7. The results of sensitivity analysis for the preterm birth less than 37 weeks outcome.**

1. Forest plot before studies exclusion. (B) Forest plot for sensitivity analysis after studies exclusion.

SRP: sub- and supra- gingival scaling and root planing; TP: tooth polishing and plaque control; ET: extraction of hopeless teeth; CR: chlorhexidine rinsing; AOR: adjustment of overhanging restorations; ST: sonic toothbrush; Mouthrinse: containing cetylpyridinium chloride; Control: the control group received no treatment or oral hygiene instruction (OHI) or oral examination (OE).
